# Evaluation of Starch-Derived Hydrogel Systems for Artifact-Cleaning Applications

**DOI:** 10.3390/gels12060557

**Published:** 2026-06-20

**Authors:** Nicola Razza, Maduka L. Weththimuni, Matteo Ferretti, Alessandro Girella, Barbara Vigani, Pietro Galinetto, Maurizio Licchelli

**Affiliations:** 1 Research Centre for Cultural Heritage (CISRiC), University of Pavia, Via A. Ferrata 3, 27100 Pavia, Italy; nicola.razza01@universitadipavia.it (N.R.); alessandro.girella@unipv.it (A.G.); 2 Department of Chemistry, University of Pavia, Via T. Taramelli 12, 27100 Pavia, Italy; matteo.ferretti02@universitadipavia.it; 3 Centre for Colloid and Surface Science (C.S.G.I.), Pavia Unit, Department of Chemistry, University of Pavia, Via T. Taramelli 16, 27100 Pavia, Italy; 4 Department of Drug Sciences, University of Pavia, Via T. Taramelli 14, 27100 Pavia, Italy; barbara.vigani@unipv.it; 5 Department of Physics, University of Pavia, Via Agostino Bassi, 6, 27100 Pavia, Italy; pietro.galinetto@unipv.it

**Keywords:** starch, hydrogels, cross-linking, SEM-EDS, µ-FTIR mapping, µ-Raman mapping

## Abstract

The demand for sustainable, high-performance biomaterials has driven intense research towards natural polysaccharide hydrogels. Accordingly, this study aimed to synthesize novel starch-based hydrogel materials, considering their inherent hydrogel-forming capabilities together with diverse potential applications (e.g., pharmaceuticals, medicine, and the cleaning application for the artifacts). To obtain hydrogels with enhanced mechanical and physico-chemical properties, starch was combined with other polymeric species (i.e., alginate, polyvinyl alcohol, and polyvinylpyrrolidone), and a gelling process was induced by using calcium cations or borate anions. Two distinct hydrogels (named S-Ca and S-SB, respectively) were prepared and characterized by a range of instrumental and experimental techniques. The assessed properties included water and solvent resistance, equilibrium water content, water-releasing capacity, morphology and microstructural features with their composition by SEM-EDS analysis, and mechanical properties (tensile strength, elasticity, Young’s modulus, and hardness). The results indicated that the investigated hydrogels exhibited suitable properties for a variety of applications, including surface cleaning processes in the field of cultural heritage conservation. For instance, they showed equilibrium water content (between 80 and 90%) comparable with other hydrogels commonly used as cleaning tools (e.g., agar and p(HEMA)/PVP) and quite low water-releasing capacity (between 10 and 17 mgcm^−2^). Moreover, the S-SB hydrogel displayed distinctly better tensile strength and elongation at break than hydrogel prepared in the presence of Ca^2+^ (S-Ca). Notably, S-SB experienced considerable elasticity improvement after freezing–thawing cycles, as indicated by a decrease in tensile strength (from 275 to 102 kPa) and an increase in elongation at break (from 121 to 275%). However, it should be noted that the hydrogel selection depends on the requirements of the target application, as different processes demand materials with distinct characteristics. Hence, both S-Ca and S-SB hydrogels were tested as cleaning tools for the removal of artificially aged acrylic coating (i.e., Paraloid B-72) from the surface of marble and wood specimens, respectively. The tests provided positive results, as aged coating was satisfactorily removed by applying the hydrogels loaded with a nanostructured emulsion (NSE). These novel starch-based hydrogels demonstrate significant potential as high-performance alternatives to conventional hydrogel systems currently used in conservation science as well as in other industrial applications.

## 1. Introduction

The development of natural products aimed at reducing or replacing synthetic materials has emerged as an important area of research. Consequently, research efforts have increasingly concentrated on sustainable materials, also referred to as ‘green materials’. For instance, the production of materials based on natural polysaccharides has greatly increased due to their biocompatibility, low cost, and non-toxicity. Moreover, their range of applications has expanded in different fields such as biomedical, pharmaceutical, food, and artifact conservation. Starch is considered a particularly attractive natural material owing to its excellent biodegradability, cost-effectiveness, and solubility. Moreover, it is widely available compared to other natural polymers, as it can be readily obtained from abundant natural sources such as rice and corn. In addition to all these properties, the possibility of chemically modifying its structure contributes to its versatility and growing utilization compared to other biopolymers [1,2]. Starch is constructed by glucose monomers linked by α-1.4 glycosidic bonds and displays three reactive hydroxyl groups (one primary and two secondary OH) per glucose unit, which can act as anchor points for chemical modifications. The main modification processes that have been reported include cross-linking and esterification, which are generally exploited to induce specific property improvements such as water resistance, thermal stability, and film-forming properties. In recent years, green, nonthermal technologies such as high-pressure processing (HPP) have emerged on an industrial scale as promising approaches for physically modifying starch, significantly enhancing its physicochemical and functional properties [1]. Chemical as well as physical modifications increase the usability of this biopolymer, allowing wider application in several different areas [3]. For instance, in addition to its common use as food, other major industrial uses are as components in paper binders and adhesives, feedstock for fermentation, textiles, chemical production, and other industrial products [3].

In the last years, starch has also been investigated as a polymeric matrix for the preparation of hydrogel materials, which have been typically produced through chemical or physical methods [1,4,5]. However, a broader application of starch-based hydrogel is limited by consumption of high energy, safety, and long processing times that often lead to environmental impacts [1,5]. Consequently, the development of efficient techniques and suitable formulations for the production of stable hydrogels based on low-cost starch sources represents a key research focus [4,5].

In the past decades, the use of hydrogel materials in the conservation of cultural heritage artifacts, particularly as surface cleaning systems, has significantly increased [6,7]. Hydrogels offer several advantages, including controlled water/solvent release and minimal invasiveness, making them especially suitable for the delicate treatment of historical surfaces. Among the various materials investigated, polysaccharide-based hydrogels such as agarose, alginate, konjac glucomannan, and carrageenan have been widely adopted as eco-friendly cleaning tools. These biopolymers are valued for their biocompatibility, biodegradability, and ability to form stable hydrogel networks, which enable the selective removal of contaminants and reduce damage to the underlying substrate. Consequently, their application has become increasingly prominent in conservation science, where sustainable and non-destructive cleaning methodologies are essential. Taking all these aspects into consideration, this study was focused on developing novel hydrogel systems based on starch and on their possible application in the artifacts.

As mentioned in the literature, starch can be used for the preparation of hydrogel materials by different chemical processes [1]. However, it should be noted that while starch forms viscous solutions when dissolved in water, it is unable to provide a hydrogel upon the addition of commonly used gelling systems that activate cross-linking processes in other polysaccharide materials. Examples of gel-forming polymer matrixes that include a starch component have already been reported. For instance, gel films were produced from starch/polyvinyl alcohol (PVA) mixtures [1,8]. These hydrogels were used for different applications in the field of medicine (tissue engineering and drug delivery systems) and agriculture [1,9,10]. Despite these numerous reports, to the best of our knowledge, only one example of a starch/PVA composite gel has been described in recent years as a useful material for cleaning artifacts [11].

Therefore, we explored the preparation of novel hydrogels derived from starch, which could be potentially applied in cleaning practices. In particular, we considered polymer composites containing, as secondary components, sodium alginate, PVA (polyvinyl alcohol), and PVP (polyvinylpyrrolidone). These polymers, which have been widely used in the preparation of different hydrogel materials, were mixed with starch, and the resulting polymer blends were crosslinked by using two common gelling agents, i.e., calcium chloride and sodium borate.

Alginate is a natural polysaccharide extracted from brown seaweed (algae), consisting of β-D-mannuronic acid (M) and α-L-guluronic acid (G) units linked by 1,4-glycosidic linkage [12,13,14,15]. It undergoes instantaneous hydrogelation in the presence of divalent cations (e.g., Ca^2+^, Ba^2+^, Sr^2+^) via the “egg-box” model [15,16].

PVA is a synthetic, hydrophilic, linear polymer produced by the hydrolysis of polyvinyl acetate and it is renowned for its high tensile strength, flexibility, and excellent oxygen barrier properties. It is semi-crystalline, which allows it to form physically cross-linked hydrogels through “freeze–thaw” cycling (cryo-gelation) [17,18]. Moreover, PVA can cross-link in the presence of additional chemical cross-linkers such as sodium borate. In fact, borax hydrolyses in aqueous solution to form boric acid and borate ions that can then react with two pairs of hydroxyl groups on different PVA chains, inducing a reversible cross-linking.

PVP is also a synthetic, water-soluble, non-ionic polymer made from the monomer N-vinylpyrrolidone, and it is highly biocompatible, has low toxicity, and possesses excellent wetting properties. Moreover, in hydrogel formulations, PVP often acts as a pore-forming agent and a film-former and increases the viscosity of the precursor solution [19].

The addition of alginate to starch solution should allow the formation of hydrogels by using calcium ions. As the resulting material appears soft and brittle, PVA and PVP were incorporated to enhance the hydrogel features. PVA provides structural reinforcement to overcome the mechanical fragility of natural polysaccharides, while PVP is utilized to modulate release kinetics and improve the overall stability of the hydrogel. Various compositions were tested to identify the optimal formulation of hydrogel cleaning tools for cultural heritage artifacts.

In the second type of hydrogel, starch was used again as a primary component and blended again with both PVA and PVP to create a mixed polymer system. This mixture was then cross-linked using sodium borate, which acts as a gelling agent by forming bonds between the polymer chains.

The starch-derived hydrogels were evaluated using a comprehensive suite of analytical techniques: morphology was assessed by scanning electron microscopy (SEM); chemical composition by energy-dispersive X-ray spectroscopy (EDS), ATR-FTIR, and µ-Raman spectroscopy; and mechanical performance by TA-XT texture analysis (tensile strength, elongation, compression, and hardness). Water absorption and releasing capacity tests were also conducted.

The main goal of this study was to prepare novel starch-based hydrogels whose features are suitable for different applications and particularly for the cleaning of cultural heritage items. Therefore, the prepared hydrogels were tested as cleaning tools in the removal of an aged polyacrylate coating (i.e., Paraloid^®^ B-72, a widely used protective coating for historical buildings since 1950 [20,21,22,23,24,25,26]) from the surface of stone (e.g., Carrara marble) and wood (e.g., maple wood). Although the preparation of the investigated hydrogels included well-known polymer components and widely used gelling agents, due to their notable features, they represent novel examples of gel materials and of promising candidates for the development of effective cleaning tools in the field of cultural heritage conservation.

## 2. Results and Discussion

The S-Ca hydrogel incorporates a blend of polymeric materials, including alginate, PVA, and PVP, alongside the primary starch biopolymer. These constituents engage in extensive hydrogen bonding via their hydroxyl (-OH) and carbonyl (C=O) functional groups, which significantly enhances the properties of the final hydrogel matrix following ionic cross-linking with Ca^2+^ ions. Specifically, each component serves a strategic purpose: PVA provides structural reinforcement to mitigate the inherent mechanical fragility of starch, PVP is utilized to modulate release kinetics and improve the overall stability of the hydrogel, and alginate facilitates the ionic cross-linking process with calcium cations to solidify the matrix. Moreover, the hydrogel was prepared as thin films (thickness about 5 mm).

In the S-SB hydrogel, the presence of PVA and PVP polymers resulted in properties consistent with the previous hydrogel. Notably, within this hydrogel matrix, PVA participates in the ionic cross-linking process with borate anions. As mentioned in the experimental part (Section 4.2), this hydrogel was exposed to three F-T cycles, expecting to improve its properties.

Both polymer materials and related hydrogels were characterized by several techniques, and the corresponding results are summarized in the following sections.

### 2.1. Morphological and Chemical Compositional Analyses (SEM-EDS)

Morphological and microstructural features of the novel hydrogels together with their chemical compositions were examined by SEM-EDS analyses performed on lyophilized samples. Moreover, micro-porosity of the hydrogel matrix was evaluated using obtained SEM images by using the analysis of FIJI software (ImageJ 1.54p, Java 1.8.0_322 (64-bit)).

**Figure 1:**
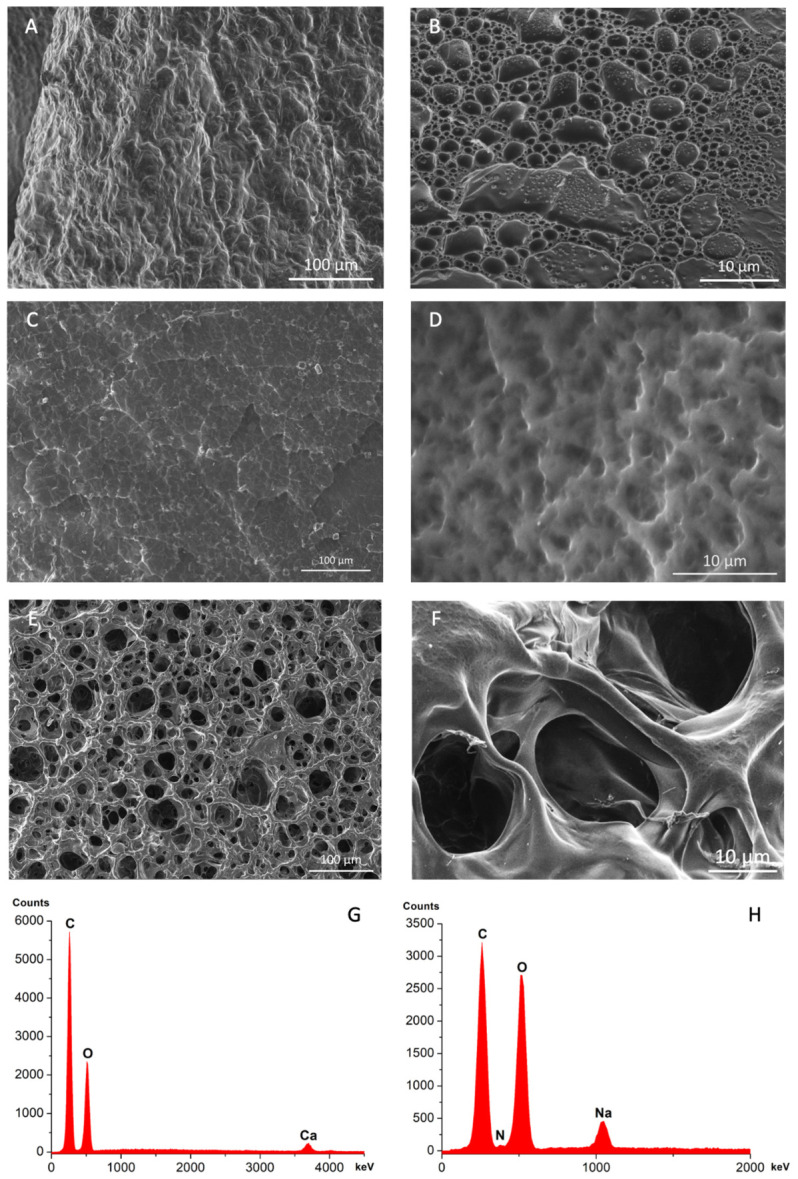
SEM-EDS analyses of dehydrated hydrogels: (**A**,**B**) SEM images of S-Ca at different magnifications; (**C**,**D**) SEM images at different magnifications of S-SB before F-T cycles, at zero cycles and (**E**,**F**) after 3 cycles; (**G**,**H**) EDS spectra of S-Ca and S-SB, respectively.

SEM observations on S-Ca showed a compact morphological structure and a poorly porous matrix (Figure 1A,B) with a rough and “groove-like” surface structure (Figure 1B).

Morphological properties of S-SB were examined both before and after the F-T cycles. The hydrogel obtained by the original polymer blend displays a rather compact and poorly porous structure (Figure 1C,D), which undergoes significant variation after repeated F-T treatment, resulting in a well-defined porous structure (Figure 1E,F).

The elemental composition of both hydrogels (S-Ca and S-SB) was assessed by EDS analysis (Figure 1G,H), which confirmed the presence of the expected elements: For instance, the presence of the element Ca (calcium ions used in the gelation process) in addition to the main components (C and O) in the case of the S-Ca hydrogel and, in the other case, the presence of C and O (from polymer backbones), N (from PVP components), and Na (from sodium borate used as a gelling agent, Figure 1H). It should be noted that a clear signal of boron, which is expected based on the composition of sodium borate, cannot be detected due to the limit of the EDS apparatus toward low-atomic-number elements.

**Figure 2:**
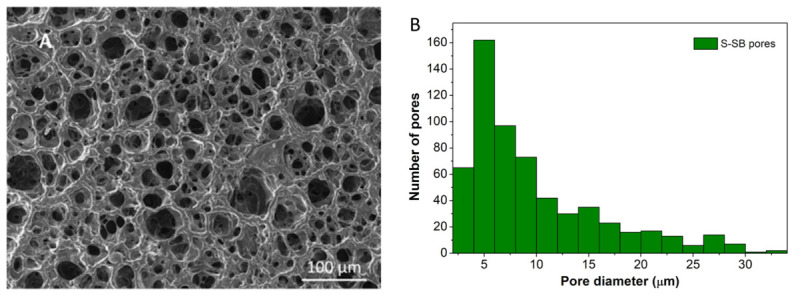
Micro-porosity calculations of S-SB hydrogel using FIJI software: (**A**) SEM image processed; (**B**) bar graph of porosity distribution.

SEM images were also used to evaluate micro-porosity [27,28,29] only for the S-SB hydrogel, as S-Ca did not display the presence of well-defined pores in the SEM observation. SEM images (Figure 2A) were processed by using FIJI software. Pore size distribution is graphically represented in Figure S1, which also reports formulas used for calculation of average pore size value 5.1 μm. The porosity distribution is also depicted in Figure 2B.

### 2.2. Mechanical Properties of Hydrogels

Mechanical analysis of hydrogel materials is an important parameter to determine their strength, elasticity, and hardness.

**Figure 3:**
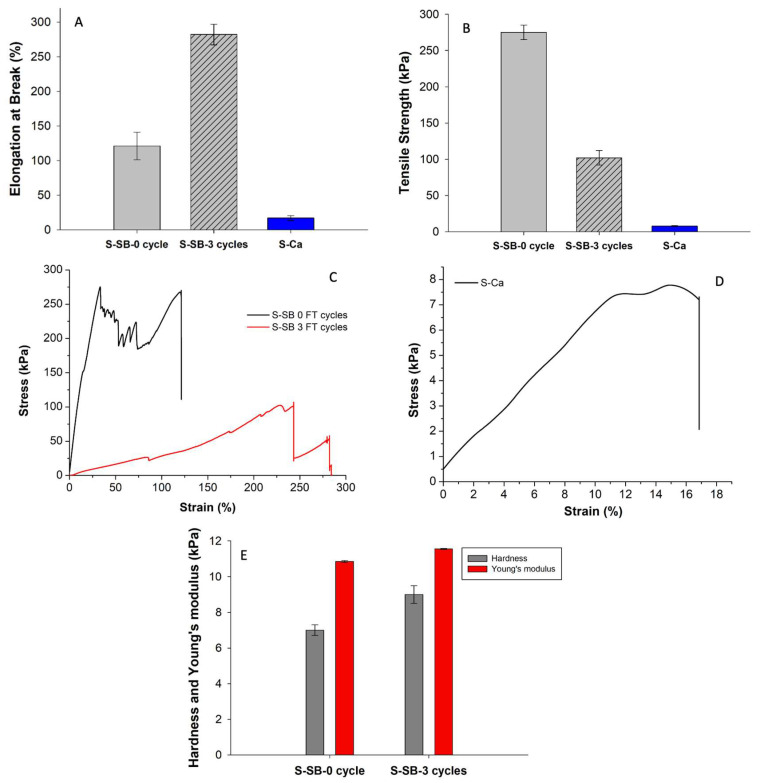
Rheological and mechanical characteristics of both S-Ca and S-SB hydrogels (for S-SB, before and after F-T cycles:3 cycles): (**A**) elongation at break; (**B**) tensile strength; (**C**) stress–strain curves of S-SB; (**D**) stress–strain curve of S-Ca; (**E**) hardness and Young’s modulus of S-SB. Mean and standard deviation values were calculated using 3 replicates.

Regarding the hydrogel cross-linked by borate anions (S-SB), it was exposed to three different F-T cycles to enhance its mechanical rearrangements. Analysis was done before and after F-T cycles (three cycles and each cycle with an exposure time of 24 h) to compare the changes due to these cycles. Tensile strength of the hydrogel decreased while exposed to F-T cycles from 275 kPa to 102 kPa, and at the same time, EB increased from 121% to 282% (Figure 3A,B). The enhancement of S-SB elastic properties induced by F-T cycles was further confirmed by observing the related stress–strain graphs (Figure 3C). Furthermore, hardness and Young’s modulus evaluated by compression tests also underwent a significant increase after exposing S-SB to three consecutive F-T cycles (Figure 3E). It is possible to illustrate the improvement of the mechanical properties considering the rearrangement of the complex polymer matrix during the F-T cycles, which provide a more defined and porous structure.

The S-Ca hydrogel has a definitely lower tensile strength (TS~5 kPa) and percentage of the elongation at break (EB~17%), when compared to the other hydrogel type (S-SB). It means that the S-Ca hydrogel has considerably lower elastic properties, as also confirmed by the related stress–strain curve (Figure 3D). Differently from S-SB, hardness and Young’s modulus were not obtained for S-Ca due to the difficulty of preparing a hydrogel sample suitable for the compression test. In fact, the ionic gelation process induced by calcium ions provides structurally heterogeneous materials when the thickness increases excessively [30]. As a consequence, S-Ca can be prepared as thin films while uniformly gelled bulk samples cannot be obtained.

The remarkable difference of mechanical properties exhibited by the two prepared hydrogels may be ascribed, in principle, to their chemical structures and/or to the crosslinking processes. As both S-Ca and S-SB contain PVA and PVP as additional polymer components, it can be supposed that the main role in determining their mechanical properties is played by the cross-linking process rather than the chemical composition. Calcium ions induce a physical gelation process in S-Ca via solely electrostatic interactions, while sodium borate in S-SB forms covalent bonds between polymer chains, which can provide improved mechanical properties, particularly with regard to elasticity.

The investigated S-Ca and S-SB have also been compared with a couple of hydrogels that are reported as cleaning tools in conservation practices, namely agar [20,31] and p(HEMA)/PVP [32], which can be considered as control materials. The mechanical properties of S-Ca are more similar to agar (TS ~13 kPa, EB ~12%) [20], which has quite a brittle behavior. On the contrary, the S-SB has a tensile strength comparable to p(HEMA)/PVP and is even more elastic than it (TS ~100, EB~150%) [20,32]. However, it should be noted that hydrogels displaying very different mechanical properties can be suitable for specific applications and, when cleaning procedures are considered, gels with very different elastic behaviors can be applied depending on the features of the surface to be cleaned.

Both morphological and mechanical analyses confirmed that, after F-T cycles, the S-SB hydrogel improved its properties. Hence, this hydrogel was used for the following characterization and the hydrogel application process.

### 2.3. Moisture Properties of Hydrogels

Water-absorption capacity (or equilibrium water content, EWC%) and water-releasing capacity (RC) are particularly significant to assess the behavior of hydrogels before using them for different applications. For instance, limited water-release behavior is required for certain applications, such as the cleaning procedures involving very sensitive surfaces.

Table 1 and Table S1 shows the results obtained from both EWC% and RC analyses. The same parameters are reported for agar and p(HEMA)/PVP hydrogels, which are taken as control materials.

S-Ca and S-SB hydrogels showed a quite similar water content at equilibrium conditions (EWC% in Table 1), while a moderately lower water-release behavior (RC in Table 1) was shown by S-Ca. When the moisture properties of novel hydrogels are compared to agar and p(HEMA)/PVP, the following considerations can be made: (i) the EWC% of S-Ca and S-SB is comparable with p(HEMA)/PVP and lower than agar; (ii) S-Ca displays a water-release behavior close to p(HEMA)/PVP, while RC measured for S-SB is slightly higher than p(HEMA)/PVP and distinctly lower than agar [20].

### 2.4. FTIR (ATR Mode) and µ-Raman Spectra

FTIR and Raman analyses contribute to a better understanding of the chemical modifications that occur during the hydrogel preparation of natural polymers. They also help assess whether hydrogel residues are released onto surfaces during the application process. Together, these techniques offer complementary insights into both the bulk composition of the hydrogels and any potential residues left on treated surfaces, which is especially relevant for conservation applications where material compatibility and surface cleanliness are critical.

**Figure 4:**
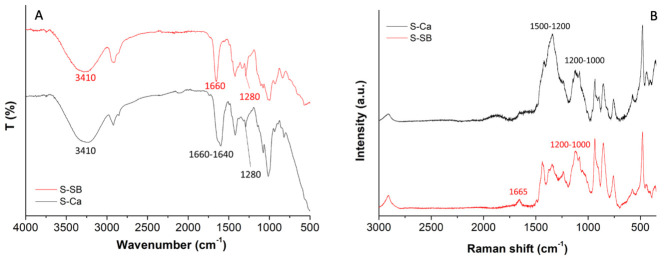
Spectroscopic analyses of prepared starch-based hydrogels (S-Ca and S-SB): (**A**) ATR-FTIR; (**B**) µ-Raman.

ATR-FTIR and µ-Raman spectra of S-Ca and S-SB (dried materials) are reported in Figure 4. With reference to the material cross-linked with calcium cations (S-Ca), the FTIR spectrum (Figure 4A) differs significantly from the spectra of the precursor polymers (Figure S2). Shifts and variations in IR signals indicate chemical modifications resulting from the blending of different polymers and from the cross-linking process. Specific observations include a broadening of the peak at 3410 cm^−1^, suggesting an increased presence of -OH groups, primarily attributed to the addition of PVA; a broad peak appearing at approximately 1640 cm^−1^ that overlaps the original asymmetric stretching vibration of COO groups (band at 1635 cm^−1^), which reflects the modifications undergone by the carboxyl groups of alginate directly involved in the interactions with Ca^2+^ ions [6,33]; the emergence of a new peak near 1660 cm^−1^ is likely due to the carbonyl stretching from PVP, while the broadening and appearance of new peaks at about 1280 cm^−1^ confirm modifications to -C-N vibrations of PVP [34]. Additionally, changes in the polysaccharide backbone can be suggested by the significant changes (mainly broadening) occurring in the 1200–900 cm^−1^ region (Figure 4A), often referred to as the carbohydrate fingerprint region, which is dominated by C-O, C-C, and C-O-C stretching vibrations associated with glycosidic linkages in the starch backbone. Within this region, bands at approximately 1047 and 1022 cm^−1^ are particularly significant, as they are related to the ordered (crystalline) and amorphous structures of starch, respectively (Figure S2) [35,36,37].

The material prepared with sodium borate (S-SB) also showed some changes in the original chemical structure of the starch biopolymer due to the ionic crosslinking process and new H-bonding interactions that can occur with the other polymers (PVA and PVP). The main changes in the FTIR spectrum (Figure 4A, S-SB) can be noted as follows: the appearance of sharp peak at around 1660 cm^−1^ (due to the C=O stretching from PVP); the appearance of a novel peak at 1280 cm^−1^ (due to the C-N stretching vibration of PVP) [34]; and the broadening of the peaks in the 1200–900 cm^−1^ range (as explained in the S-Ca hydrogel, it can be due to the C-O, C-C, and C-O-C stretching vibrations corresponding to glycosidic linkages in the starch polymer backbone). All these changes can be attributed to the chemical modifications that happened in the hydrogel matrixes.

The µ-Raman results (Figure 4B) also indicate that the original chemical structures of considered biopolymers changed due to various new interactions (e.g., H-bonding) between the different macromolecular components and also the cross-linking processes as already indicated by the FTIR results. For instance, the spectrum of S-Ca showed several differences when compared to the spectra of the original polymers (e.g., starch, Figure S3): for instance, the very broad band observed in the region between 1500 and 1200 cm^−1^ can be explained by the convolution of various vibration signals (CH and CH_2_ bending, C-O and C-C stretching, C-CH, C-OH, and C-CH deformations) [38,39], which are very poorly resolved due to the complexity of interactions within the polymer matrix. Similar considerations can be made on the broad absorption in the 1200–1000 cm^−1^ spectral region, where some characteristic peaks due to C-O and C-C stretching and C-O-C deformation are expected, in particular those referring to the α-glycosidic bonds (amylose and amylopectin chains in starch), as well as to the guluronic (band at approximately 1025 cm^−1^) and the mannuronic units (band at approximately 1100 cm^−1^) in alginate [38,39,40].

The µ-Raman spectrum of S-SB (Figure 4B) showed the clear appearance of the carbonyl peak of PVP at around 1665 cm^−1^, and some changes of the peaks in the range between 1200 and 1000 cm^−1^ due to the changes can happen in the skeleton of the main polymer (starch) as explained in the S-Ca hydrogel. Anyway, the pronounced broadening of signals in the 1500–1200 cm^−1^ range observed in the case of S-Ca is not observed for S-SB, suggesting that it was mainly caused by the presence of alginate and by the strong electrostatic interactions that take place when cross-linking with calcium ions is carried out.

### 2.5. Hydrogel Applications and Cleaning Assessment

Both novel hydrogels were applied to examine their performance as cleaning tools for artifacts. For this purpose, as explained in the experimental section (Section 4.4), two different types of mockups (Carrara marble and maple wood) were prepared and coated with Paraloid B-72 polymer. The coated mockups were artificially weathered according to the previously reported method (temperature = 70 ± 2 °C for 35 days) [20] to obtain the aged coating that simulates real cases. The hydrogel samples were loaded with nanostructured emulsion [20], and they were applied on the surfaces of aged, coated mockups to evaluate the ability to remove decayed coating from the surfaces of marble and wood. The two types of hydrogels were used in different ways depending on their moisture properties and mechanical behavior. In particular, the S-Ca hydrogel, which displays lower retention behavior, was applied on wood mockups, considering that wood surfaces are more sensitive toward water and need lower exposure to water as well as water-containing cleaning fluids. On the contrary, S-SB that displays a larger water release was applied to marble mockups.

The cleaning performance of both investigated hydrogel tools was evaluated using several analytical techniques, including SEM, chromatic variation analysis, contact angle measurements, µ-ATR-FTIR, and µ-Raman mapping.

SEM analyses were carried out to examine the morphological features of stone and wood surfaces before and after the cleaning process. Moreover, hydrogel films (S-Ca and S-SB, non-lyophilized) were examined by the SEM technique to understand the morphological differences of hydrogel structure before and after cleaning applications. As shown in Figure S4, both hydrogels substantially retained morphological features like their original structure (Figure S4A,C) even after cleaning (Figure S4B,D), suggesting that their structural integrity was preserved and that they can be reused multiple times.

**Figure 5:**
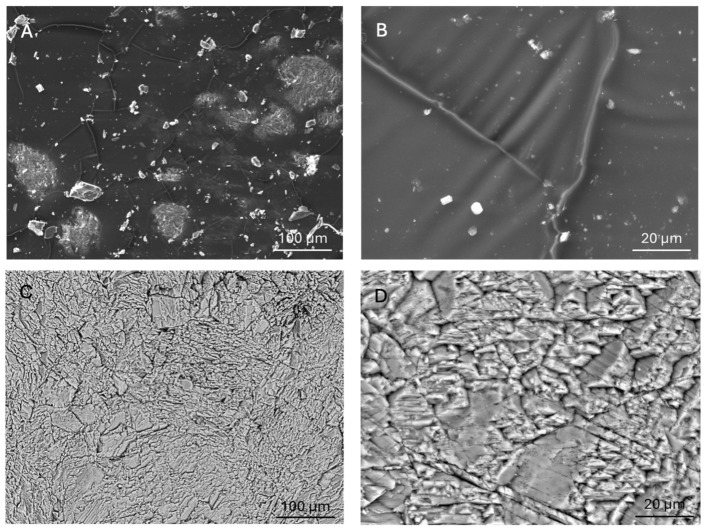
SEM images of Paraloid B-72-coated marble specimens after aging (**A**,**B**) and after application of S-SB cleaning system (**C**,**D**).

Before cleaning, the layer of artificially aged Paraloid B-72 was clearly observed over the surface of the marble mockup (Figure 5A,B). The polymer coating showed some cracking and partial melting phenomena as possible results of aging, while the uncoated surface of stone appeared only in limited areas. After the application process, the marble surface appeared free from any residual coating, indicating the satisfactory removal of weathered polymer (Figure 5C,D).

**Figure 6:**
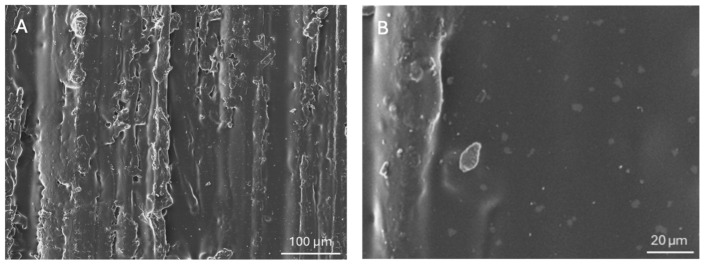

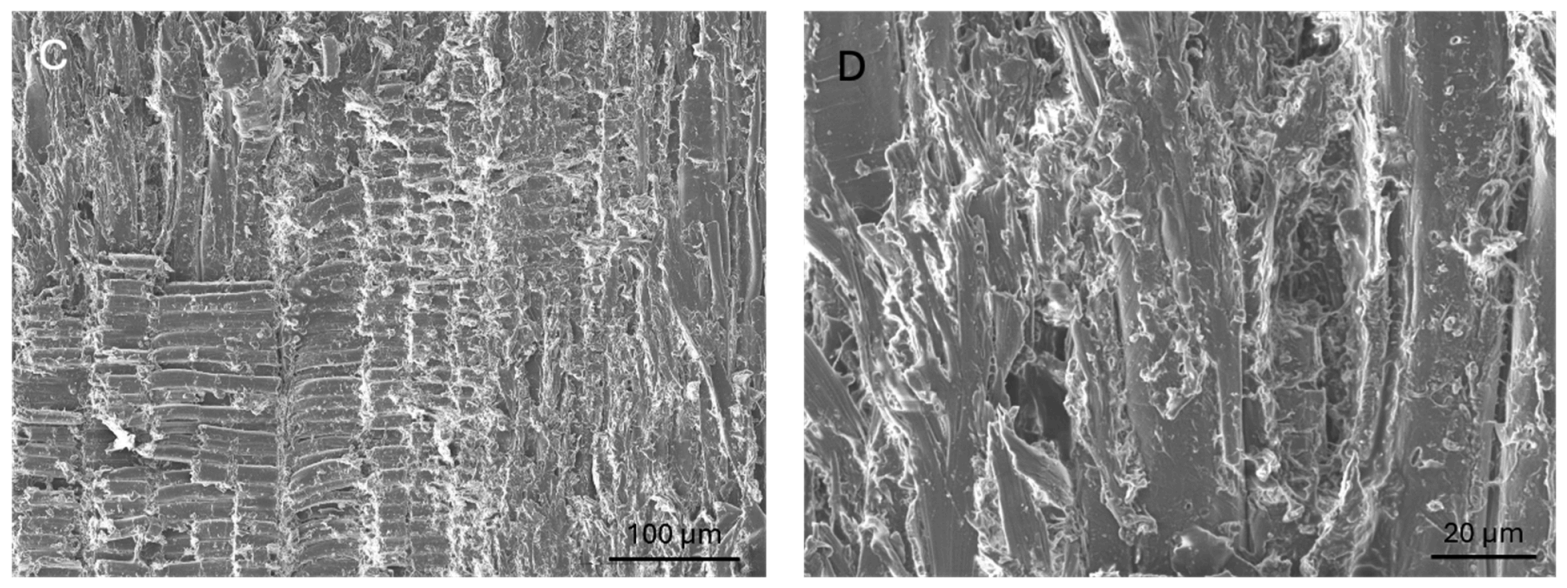
SEM images of the Paraloid B-72-coated wood mockups after aging (**A**,**B**) and after hydrogel (S-Ca) cleaning processes (**C**,**D**).

In the wood mockups, similar morphological differences were observed, with the surface being fully covered before the cleaning process and the natural wood morphology becoming visible after removal of the polymer layer (Figure 6).

Chromatic variations measured on both marble and wood mockups coated with Paraloid B-72, after exposure to the aging cycle and following the hydrogel cleaning process, are summarized in Table 2 and Table S2. Before cleaning, large overall chromatic variations were measured (ΔE* = 5.3 and 17.9 for marble and maple, respectively). In the case of the stone surface, the L* was mainly affected (ΔL* = −5.3) by the presence of the aged coating, likely due to the enhanced brightness induced by the polymer layer. Considering the wood surface, a large variation was observed for the b* coordinate (Δb* = 5.8), likely due to the yellowing of the weathered coating, in addition to a significant variation in brightness (ΔL* = −5.7).

**Figure 7:**
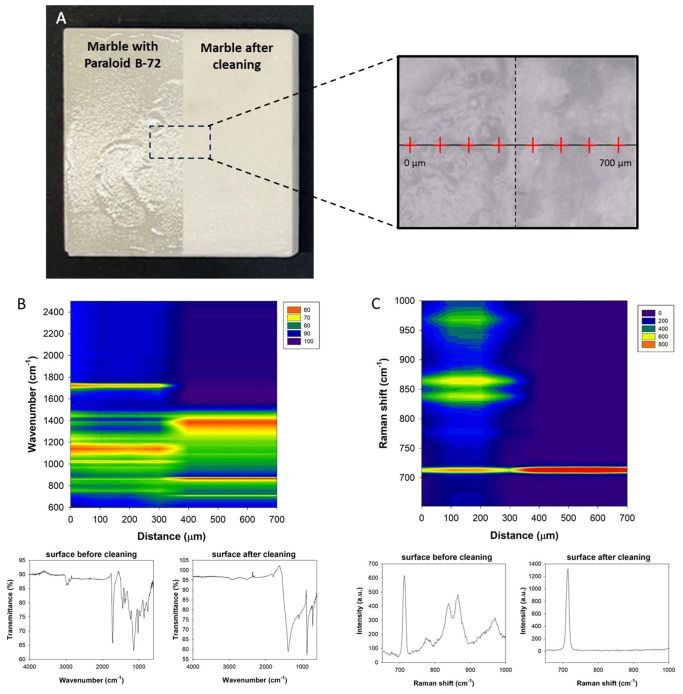
The images of the Carrara marble surface before and after removing the aged polymer coating (**A**), its μ-ATR-FTIR (**B**) and μ-Raman mapping (**C**). Single FTIR and Raman spectra taken before the application of the hydrogel tool and after the cleaning process are also reported.

Cleaning of the coated marble surface using the NSE-loaded S-SB hydrogel resulted in a significant reduction in the overall chromatic variation from 5.3 to 1.4. It indicated that the marble surface returned to exhibit a chromatic aspect very close to its original one (Figure 7A), undergoing an overall color variation that cannot be identified by the naked eye (∆E* < 5) [41,42].

Application of the NSE-loaded S-Ca hydrogel on the surface of the coated wood specimens provided similar, quite satisfactory results: the decrease in the overall chromatic variation was very substantial (ΔE* reduced from 17.9 to 3.9), suggesting an effective cleaning process and a surface condition close to that of natural uncoated wood.

The cleaning performance of hydrogels was also evaluated by performing contact angle measurements (Table 3 and Table S3) on the coated mockups before and after hydrogel application. Before cleaning, both mockups exhibited hydrophobic surfaces (α_marble_ = 115° and α_wood_ = 108°), as expected by the presence of the water-repellant polyacrylate coating. After cleaning, the contact angle of the marble considerably decreased (from 115 to 88°), in agreement with the removal of the polymer layer. It should be noted that the plain marble has a contact angle around 81°, which is quite similar to a cleaned marble surface.

In the case of the wood surface, it was not possible to measure the contact angle after performing the cleaning procedure, as expected for a complete removal of the Paraloid B-72 coating, which made the surface become again highly hydrophilic.

**Figure 8:**
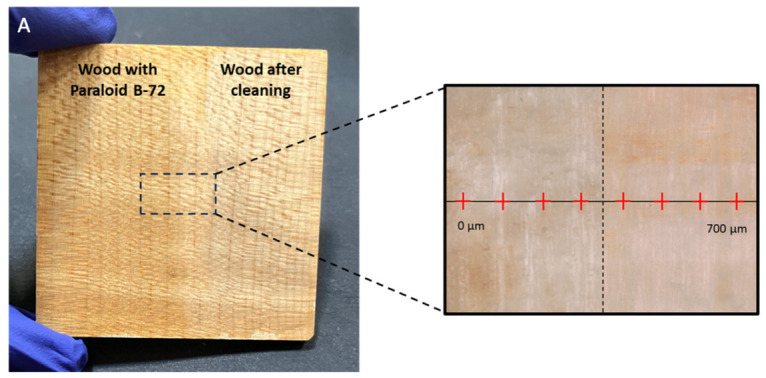

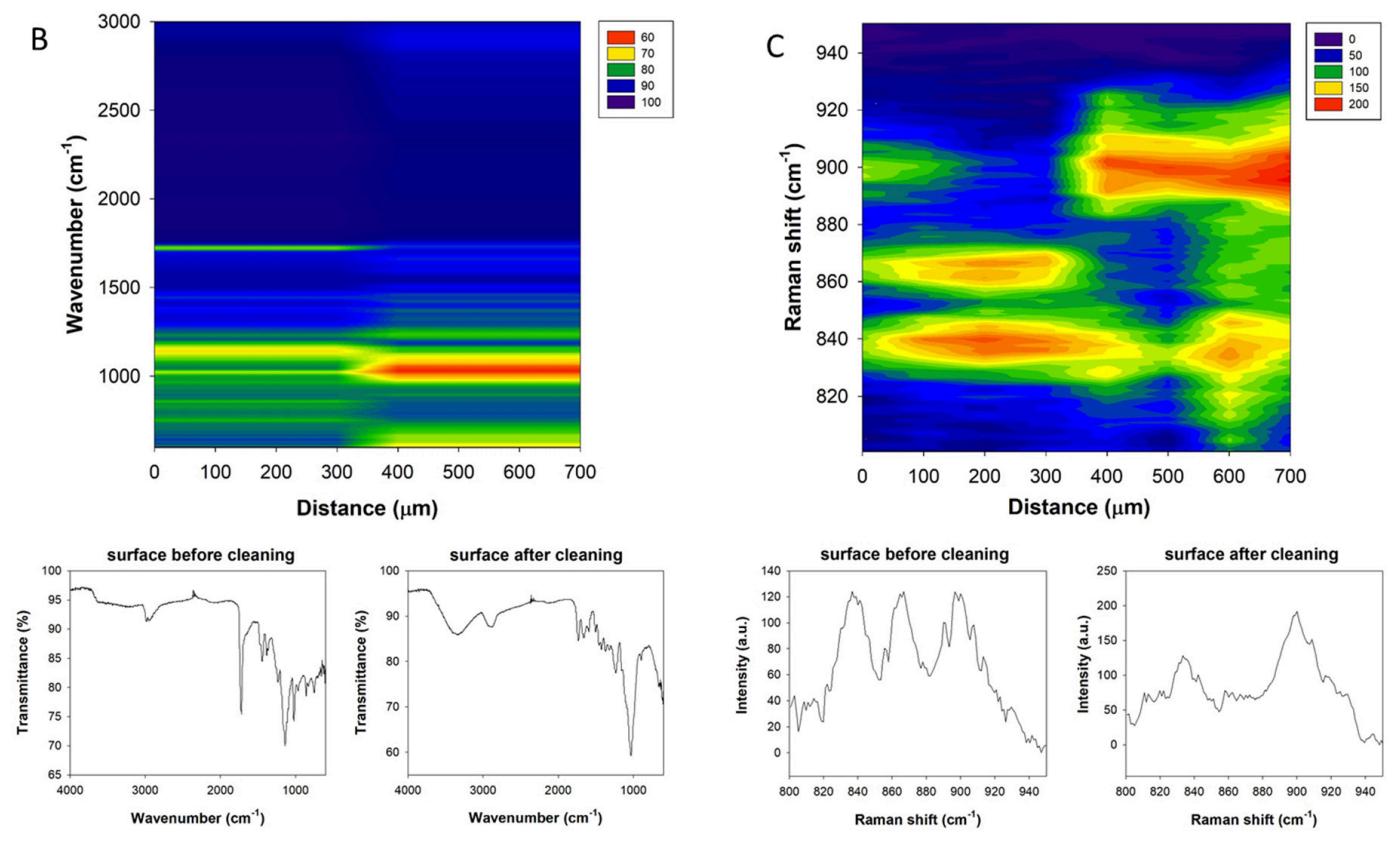
The images of the coated maple wood surface before and after removing the aged polymer coating (**A**), its μ-ATR-FTIR (**B**) and μ-Raman mapping (**C**). Single FTIR and Raman spectra taken before and after cleaning are also reported.

Moreover, µ-ATR-FTIR and µ-Raman mapping experiments were involved in investigating in detail the cleaning process: the chemical composition of the stone and wood surface was examined before and after applying the polymer-removing tools. The main results are summarized in Figure 7 and Figure 8. The examination was done on a one-side-treated surface of stone and wood mockups (left side of the mockups in Figure 7A and Figure 8A) and a cleaned one (right side). The optical microscope images of the surface areas studied by mapping experiments are also reported in the same figures. Spectra were collected every 100 µm until 700 µm. The first spectra were collected on the polymer-treated surface (from 0 to 300 µm), while the other reported spectra were collected on the cleaned surface by hydrogel loaded with nanostructured emulsion (from 400 to 700 µm).

The FTIR mapping performed on the marble surface (Figure 7B) confirmed the presence of acrylic polymer (Paraloid B-72) on the uncleaned surface area. In particular, the sharp peaks at around 1750 and 1150 cm^−1^ (related to C=O and C-O stretching, respectively) [43,44] were clearly observed in the spectra taken on the coated area. After application of the cleaning tool based on the S-SB hydrogel, representative peaks of Paraloid B-72 completely disappeared, while three main peaks at 1450, 875, and 670 cm^−1^ were observed instead in the spectra taken on the cleaned area. These absorptions are ascribed to calcium carbonate [45,46], which is the main, if not unique, component of Carrara marble, verifying that Paraloid B-72 was entirely removed from the treated surface. Similar results were obtained from µ-Raman mapping experiments (Figure 7C), which confirmed the cleaning performances of the novel hydrogel tool. In particular, two specific peaks that are due to Paraloid B-72, observed in the spectra of the uncleaned area at around 836 and 863 cm^−1^ [47,48,49], disappeared when the spectra taken on the cleaned area were considered. In fact, after the application of the S-SB hydrogel, only a peak at 712 cm^−1^ was observed, which is related to calcite as expected from the chemical composition of Carrara marble.

The false-color maps (Figure 7B,C) graphically display the data obtained from the FTIR and Raman spectra.

A similar investigation was performed on wood mockups (Figure 8A–C). Before the cleaning process, µ-FTIR mapping showed the acrylic polymer coating on the surface of the wood mockups, mainly indicated by the sharp carbonyl band at around 1750 cm^−1^, together with other specific peaks related to the polymer [43,44]. No significant peaks attributable to natural wood were observed, indicating that the polymer completely covered the wood surface.

In contrast, after the application of the (S-Ca) cleaning tool, only peaks characteristic of natural wood appeared in the spectra, demonstrating the entire elimination of the Paraloid B-72 from the maple mockups. For instance, the broad and intense peaks of cellulose (1162, 1110 and 1035 cm^−1^) are clearly visible in the FTIR mapping [50,51].

As also observed in the Raman spectra of the marble stone, two specific bands related to Paraloid B-72 can be noted at around 836 and 863 cm^−1^, together with some peaks attributable to natural wood (e.g., cellulose). After the hydrogel cleaning treatment, the complete disappearance of the polymer peaks and the appearance of cellulose signals were observed, especially the prominent peak at 898 cm^−1^ [52,53], which is clearly identifiable in the Raman mapping.

Cleaning tests performed on marble and wood surfaces clearly showed that aged polymer coating can be efficiently removed by applying films of S-SB and S-Ca loaded with a nanostructured fluid, respectively. Moreover, it is worth noting that no hydrogel residues were evidenced on the surface of cleaned marble and wood mockups, as demonstrated by the absence of IR and Raman signals ascribable to the investigated hydrogels (Section 2.4) during the mapping experiments.

Taking into consideration the control hydrogel p(HEMA)/PVP, which is commonly used for artifact-cleaning applications, it showed complete removal of polymer coatings (e.g., acrylic polymers and vinyl polymers), as well as the removal of adhesives from different substrates, which was examined and confirmed using the FTIR spectroscopic technique [54,55,56]. It indicates that the hydrogels, even when loaded with the envisaged nano-emulsion (mixture of water, 1-butanol, and butanone), possess good mechanical strength and well-structured matrices suitable for the application on stone as well as wood surfaces. These novel hydrogel systems can therefore be proposed as promising alternatives to conventional hydrogel materials, which often present several limitations when applied in the conservation of artwork as well as in other various applications.

### 2.6. Results of the Durability Analysis of Hydrogels

The durability analysis is an important factor in evaluating both the reusability and long-term degradation behavior of hydrogels. The durability of the investigated hydrogels was assessed by examining S-Ca and S-SB old samples that were prepared, dried, and stored at room temperature (RT = 22 ± 3 °C) in the laboratory.

**Figure 9:**
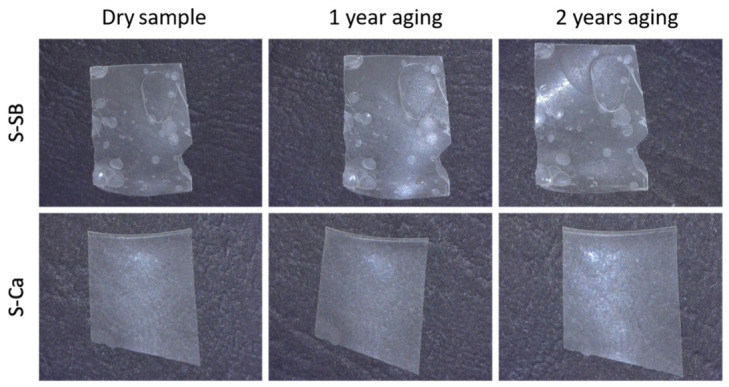
Digital microscope images during the durability analysis of S-SB and S-Ca hydrogels.

Since their preparation (more than 2 years ago), the samples have been regularly observed by digital microscope (Dino-Lite) to observe their aspect and any possible microbial growth. Figure 9 reports selected images taken during the durability analysis, particularly after about 1 and 2 years. The results indicate that the dried hydrogel films (both S-SB and S-Ca) did not undergo significant modifications (for instance, no syneresis phenomena). Moreover, no changes related to natural microbial growth were observed even after 2 years from preparation, indicating a strong resistance to microbial decay.

## 3. Conclusions

This study primarily aimed to synthesize and thoroughly characterize new hydrogel materials based on biopolymers, particularly starch. The obtained results can be summarized as follows:

(1) Two distinct hydrogels were successfully developed through cross-linking of the starch-containing polymer matrices using calcium cations (the obtained hydrogel was named S-Ca) and borate anions (the hydrogel was named S-SB) as gelling agents and alginate, PVA, and PVP as additional polymeric components.

(2) The results revealed some relevant differences between the two systems. For example, the S-SB hydrogel showed higher moisture-related properties than S-Ca, with an equilibrium water content (EWC) of approximately 86% and 83%, respectively, and a water-releasing capacity (RC) of about 16 and 12 mg cm^−2^, respectively. More pronounced differences were observed in their mechanical behavior. The tensile strength (TS) of the S-Ca hydrogel was around 5 kPa, while the S-SB hydrogel reached approximately 102 kPa. Similarly, the elongation at break (EB) increased from about 17% in S-Ca to nearly 282% in S-SB, indicating a significantly enhanced elasticity.

(3) Despite these differences, both types of hydrogels demonstrated unique and valuable characteristics, making them potentially suitable for different applications. This versatility was highlighted through their successful use in two distinct cleaning applications. For instance, aged Paraloid B-72 polymer coating was entirely eliminated from the surfaces of Carrara marble and maple wood mockups by S-Ca and S-SB, respectively, which were loaded with a nanostructured emulsion as cleaning fluid.

(4) The complete removal of aged Paraloid B-72 polymer without releasing any residues was clearly evidenced and confirmed by several instrumental techniques: the differences observed in overall chromatic variation before and after the removal of the polymer coating (for marble, the ΔE* value changed from 5.3 to 1.4, and for wood, from 17.9 to 3.9), indicating that the surfaces returned close to their original appearance; changes in hydrophobic properties (the wood surface became highly hydrophilic due to the removal of the polymer layer, while the marble surface also showed reduced hydrophobicity); and both µ-ATR-FTIR and µ-Raman mapping demonstrated that the polymer coating was completely removed from the surfaces without leaving any residues.

(5) In fact, all these results demonstrate that these novel starch-based hydrogels can be proposed as effective and adaptable materials in conservation practices.

## 4. Materials and Methods

### 4.1. Materials

Starch from rice (C_6_H_10_O_5_)_n_ (S, Mw of the monomer: 162.14 gmol^−1^) and medium-viscosity sodium alginate (C_6_H_7_NaO_6_)_n_ (Alg, Mw~80,000–120,000 gmol^−1^, Mw of the monomer: 198.11 gmol^−1^) were purchased from Merck Life Science S.r.l. (Milan, Italy). Anhydrous calcium chloride (CaCl_2_) was supplied by Fluorochem Ltd. (Hadfield, UK). The nano-emulsion was prepared using the non-ionic surfactant ECOSURF^TM^ EH-6, C_8_H_18_O·(C_3_H_6_O)x·(C_2_H_4_O)y (Sigma Aldrich, St. Louis, MO, USA), 2-butanol (99% BuOH, C_2_H_5_CH(OH)CH_3_, Fluka Chemicals, Buchs, Switzerland), and 2-butanone (99–101% CH_3_C(O)CH_2_CH_3_, BDH Chemicals Ltd., Poole, UK). Polyvinylpyrrolidone (C_6_H_9_NO)_n_ (PVP) and polyvinyl alcohol (CH_2_CH(OH))_n_ (PVA) were supplied by Sigma Aldrich (St. Louis, MO, USA). Sodium tetraborate decahydrate (Na_2_[B_4_O_5_(OH)_4_].8H_2_O, borax) was purchased from Carlo ERBA (Cornaredo, Milan, Italy). Paraloid B-72 (100% acrylic resin) was purchased from Bresciani s.r.l (Milan, Italy).

Whatman^®^ qualitative filter paper, Grade 1 (WHA1001090), was used for the moisture property examinations. Water was purified using a Millipore Organex system: R ≥ 18 M cm (Burlington, MA, USA).

### 4.2. Hydrogel Preparation Methods

Initially, various polymer compositions were blended to determine the optimal ratios for hydrogel development. Following a preliminary analysis to evaluate these formulations, this study proceeded using two specific cross-linking methods: ionic cross-linking with calcium cations and borate anions. For instance, a starch biopolymer was modified by incorporating PVA and PVP, then cross-linked with sodium borate (designated as S-SB). Conversely, a starch polymer matrix was modified with Alg, PVA, and PVP and subsequently cross-linked with calcium chloride (named as S-Ca). The hydrogel preparation is summarized below:

S-Ca hydrogel: rice starch (0.2 g, 1.2 × 10^−3^ mol with respect to the monomer) was dissolved in 15.0 mL of water in a beaker at 90 °C, using a magnetic stirrer to mix the solution. Once a homogeneous fluid was obtained, 0.2 g (1.0 × 10^−3^ mol with respect to the monomer) of sodium alginate (Alg) powder was added directly into it. PVA (0.3 g) was dissolved in 3.0 mL of water by bringing the water to a boil in a glass vial, and it was added to the previous polymer mixture (starch and alginate polymer mixture) only when the solution appeared clear (fully dissolved in boiled water). Finally, 0.1 g (0.9 × 10^−3^ mol with respect to the monomer) of PVP is added by continuously stirring the mixture for around 30 min to rearrange the polymer chains. After 30 min, the resulting solution was subjected to an ultrasonic bath (or, alternatively, nitrogen air was fluxed) in order to remove air bubbles, and then it was poured into a mold in order to form a thin layer (thickness about 5 mm). Then, 20 mL of 2% (*w*/*v*) CaCl_2_ aqueous solution was slowly added into the mold to introduce the calcium ions that will act as a crosslinker. After about 10 min, or in any case when the film has stiffened sufficiently, it is turned upside down to facilitate the diffusion of Ca^2+^ ions also on the lower surface and kept for another 10 min. Once crosslinking was completed, the thin film obtained was washed carefully with distilled water to remove excess and unreacted calcium ions.

S-SB hydrogel: Starch (0.2 g, 1.2 × 10^−3^ mol relative to the monomer unit) was dissolved in 15.0 mL of water at 90 °C in a beaker. Then, 0.1 g (0.9 × 10^−3^ mol relative to the monomer unit) of PVP was added under continuous magnetic stirring. Separately, 0.3 g (6.8 × 10^−3^ mol with respect to the monomer) of PVA was dissolved in 3.0 mL of water by boiling in a glass vial, and it was added into the previous polymer mixture when the PVA completely dissolved. The solution was continuously magnetically agitated at around 90 °C for 30 min to obtain a homogeneous solution, and then 0.05 g (0.1 × 10^−3^ mol) of sodium borate (SB) was also added and mixed vigorously until a hydrogel was obtained. Once the hydrogel was brought to room temperature, it was subjected to three freezing–thawing cycles (F-T), in which it was left at −20 °C for 20 h and at room temperature (20 °C) for 4 h, all repeated three times (one cycle was 24 h). As reported in the literature for different PVA-containing hydrogel materials, this allows the hydrogel structure to rearrange and improve the mechanical properties [17]. After this process, hydrogel (S-SB) was poured inside into different molds to prepare thin films (thickness about 5 mm) and cubic (size about 1 cm^3^) and cylindric (volume about 6 cm^3^) hydrogel samples.

### 4.3. Characterization of Polymer and Hydrogel Materials

#### 4.3.1. SEM-EDS Analyses

The morphological properties of the prepared hydrogels were analyzed through scanning electron microscopy (SEM) employing a Tescan FE-SEM (MIRA XMU series; TESCAN, Brno, Czech Republic). Prior to analysis, samples were dehydrated using a freeze-dryer instrument (Labconco, FreeZone 2.5 plus, Kansas City, MI, USA) with the following conditions: temperature: −87 °C and vacuum (0.050 mbar) for 24 h.

In particular, the differences in the polymer matrix due to the F-T cycles were evaluated for the materials prepared by cross-linking with borate anions. Furthermore, energy dispersive X-ray spectrometry (EDS) analyses were performed using a Bruker Quantax 200 instrument (Bruker, Billerica, MA, USA) to determine the chemical composition of the new hydrogels. The SEM-EDS instrument operated in high vacuum (located at the Arvedi Laboratory, CISRiC, University of Pavia, Pavia, Italy).

Moreover, SEM images were used to calculate the micro-porosity of hydrogels by using FIJI software (ImageJ 1.54p, Java 1.8.0_322 (64-bit)), an open-source distribution of the scientific image processing package ImageJ2 [https://imagej.net/software/fiji/downloads accessed on 19 June 2026].

#### 4.3.2. Mechanical Properties

The mechanical properties (tensile strength, elongation at break, compression strength and Young’s modulus) of newly prepared hydrogels (wet films as prepared without drying) were measured and compared with commonly available physical hydrogels. For the measurements of tensile strength and elongation at break, hydrogel samples were stretched uniaxially and measured; mechanical properties were evaluated using a TA.XT Plus texture analyzer (Stable Micro Systems, Godalming, UK) equipped with a 5 kg load cell. Prior to testing, the thickness of the films was determined using a Sicutool 3955G-50 micrometer (Sicutool, Milan, Italy). Each hydrogel film specimen (1 × 3 cm) was then mounted onto an A/TG tensile grip, with an initial clamp separation of 1 cm. The upper grip was moved upward at a constant rate of 2 mm/min until a displacement of 50 mm was reached. Tensile strength and elongation at break were subsequently calculated from the acquired data. For each hydrogel formulation, measurements were performed in triplicate, and the results were expressed as mean values. Furthermore, the compressive strength and Young’s modulus of the hydrogels were evaluated. The hydrogels were cast in a cylindrical-shape matrix (30 mm diameter × 30 mm height) and subjected to compression testing using a TA.XT Plus Texture Analyzer (Stable Micro Systems, Godalming, UK) fitted with a 5 kg load cell and a P/10 measuring system comprising a cylindrical probe with a diameter of 10 mm [57]. During the test, the probe was lowered at a constant speed of 1.00 mm/s until the samples reached 70% deformation. A maximum deformation of 70% of the initial sample height was selected to ensure that the measured force reflected the mechanical resistance of the hydrogel, minimizing artifacts associated with excessive compression, sample collapse, or probe–platform contact. The following mechanical properties were evaluated: (a) hardness, defined as the maximum compressive force per unit area required to disrupt the hydrogel structure, and (b) Young’s modulus (YM), determined from the slope of the initial linear region of the compressive stress–strain curve. Stress–strain curves were generated for all samples. Each hydrogel formulation was tested in triplicate. The resulting experimental data were analyzed statistically using Statgraphics 5.0 (Statistical Graphics Corporation, Rockville, MD, USA). Differences between group means were assessed using a *t*-test, with statistical significance determined accordingly. For this purpose, GraphPad Prism software (version 10.1.1, GraphPad Software, San Diego, CA, USA) was used.

#### 4.3.3. Moisture Properties

Equilibrium water content (EWC%) and water-releasing capacities (RCs) were measured following standard protocols as previously reported [20]. In order to obtain statistical data, three replicates were used for each analysis, and finally, averages and standard deviations were calculated.

#### 4.3.4. ATR-FTIR and µ-Raman Analyses

Before doing the analysis, hydrogel films were dried under vacuum at around 20 °C for 24 h.

FTIR spectra were collected in ATR mode by using a PerkinElmer Spectrum 100 FT-IR instrument (Perkin-Elmer, Waltham, MA, USA), recorded over the spectral range of 450–4000 cm^−1^ with a resolution of 4 cm^−1^. µ-FTIR mapping analyses were done before and after cleaning the Paraloid B-72 aged polymer coating on the considered surfaces using a Nicolet iN10 µFT-IR spectrometer (Thermo Fisher Scientific, Waltham, MA, USA) operated in attenuated total reflectance (ATR) mode with a germanium crystal. A resolution of 4 cm^−1^ and a total of 64 scans were collected for each spectrum.

Collection of µ-Raman spectra and mapping experiments carried out before and after the cleaning procedure were performed using a Labram Dilor Raman spectrometer HD10, equipped with an Olympus HS BX50 microscope. Two different beams were used for stone (He-Ne laser at 632.8 nm) and wood (laser diode at 785 nm) materials. For instance, the beams were focused on the surfaces of about 2 µm diameters, and the scattered light was collected through the same microscope objective lens. The Raman spectra were recorded by a multi-channel diode array detector, and all the measurements were made at room temperature. The spectrums were accumulated in the specific spectral range (100–1900 cm^−1^) with a resolution of 2 cm^−1^. Moreover, the final spectra were obtained by averaging several spectra from a linear scan.

### 4.4. Hydrogel Applications

Performances of considered hydrogels were evaluated by applying them as cleaning tools for the elimination of aged Paraloid B-72 coating (the application of acrylic coatings and aging process were performed according to standard procedures reported elsewhere) [20] from the surface of mockups made of different materials (Carrara marble and maple wood). In both cleaning applications, thin hydrogel films (5 mm thickness of S-SB and S-Ca, respectively) were prepared to match the treated mockup surfaces (about 12.5 cm^2^) and they were partially dehydrated (vacuum at 25 °C for 1 h) and then dipped into the nanostructured emulsion (NSE: H_2_O, 65.9%; surfactant-EcoSurf^TM^, 3.5%; BuOH, 9.7%; and butanone 20.9%) [20,58] for 24 h and 4 h, respectively. After that, the hydrogel films were applied to the considered mockup surfaces for 15 min for the cleaning process. After removing both S-SB and S-Ca hydrogel films from the stone and wood surfaces, any eventual residues on the cleaned surfaces were gently eliminated using a wet cotton swab. The cleaning applications were repeated three times in order to obtain correct results. Performances of the investigated hydrogel cleaning tools were evaluated by performing color and contact angle measurements: in order to examine the chromatic variations due to the cleaning processes, the chromatic coordinates were measured on the surface of marble stone as well as maple wood specimens treated with aged Paraloid B-72 (before cleaning) and after the application of the hydrogel cleaning systems (after cleaning). All experimental measurements were performed in three replicates, and results are expressed as mean ± standard deviation. Inferential statistical analyses were carried out using Microsoft Excel to assess the efficacy and safety of the cleaning treatments, with the threshold for statistical significance set at *p* < 0.05. The measurements were done using a Konica Minolta CM-2600D spectrophotometer (Konica Minolta, Inc., Tokyo, Japan), assessing the L*, a*, and b* parameters of the CIELAB color space and the total chromatic variation (ΔE*) according to the UNI EN 15886 protocol [59]. Fifteen different measurements (three mockups for each kind of specimen and five measurements on each specimen) were performed to obtain the accurate results, and the average values and standard deviation were reported as recommended by the literature [41,50]. Moreover, for colorimetric data, a paired Student’s *t*-test was employed to evaluate the significance of chromatic shifts (ΔL*, Δa* and Δb*) on both marble and wood substrates by directly comparing the values before and after the hydrogel cleaning.

Contact angle measurements were done on the same samples used for colorimetric analyses (before and after cleaning applications) using a Lorentzen and Wettre instrument (Zurich, Switzerland) according to the UNI EN 15802 Protocol [60]. The average values were calculated using fifteen different measurements as explained in the colorimetric measurements and previously reported [41]. Moreover, for contact angle measurements on the marble substrate, a one-way Analysis of Variance (ANOVA) was performed to compare the three different surface states (untreated, with aged Paraloid B-72, and after cleaning). A Tukey’s Honestly Significant Difference (HSD) post hoc test was applied to determine specific pairwise differences between the groups and evaluate the recovery of the original substrate wettability.

Moreover, morphological differences were investigated by SEM, and the chemical modifications were examined by handling µ-ATR-FTIR and µ-Raman mapping experiments on the uncleaned/cleaned surfaces.

### 4.5. Durability Analysis of Hydrogels

The durability of the prepared hydrogels was investigated using a digital microscopic technique (Dino-Lite, Almere, Netherlands) under laboratory conditions. For this purpose, dried hydrogel samples stored in the laboratory for a long time from their first preparation (more than 2 years) at room temperature (RT = 22 ± 3 °C) were observed. At different time intervals, the samples were examined microscopically to evaluate the presence of possible microbial growth.

## Figures and Tables

**Table 1:** The moisture properties of the hydrogels (three replicates were used to determine the average values).

Hydrogels	Equilibrium Water Content (EWC, %)	Water-Releasing Capacity (RC, mg/cm^2^)
S-Ca	83.3 (±3.1)	11.9 (±2.6)
S-SB	86.3 (±2.8)	16.2 (±0.6)
Agar * p(HEMA)/PVP *	95.4 (±2.0) 81.0 (±2.0)	28.0 (±1.7) 13.0 (±2.0)

* Data from Reference [20].

**Table 2:** Chromatic variations of the Paraloid B-72-treated mockups after aging and after cleaning by NSE-loaded hydrogels (three replicates were used to calculate the average values).

Samples	With Aged Paraloid B-72				After Cleaning with NSE-Loaded Hydrogels			
	ΔL*	Δa*	Δb*	ΔE*	ΔL*	Δa*	Δb*	ΔE*
Marble	−5.3 (±0.2)	−0.1 (±0)	0.5 (±0.2)	5.3 (±0.3)	−1.3 (±0.2)	−0.1 (±0)	0.5 (±0)	1.4 (±0.2)
Wood	−5.7 (±0.3)	0.8 (±0)	5.8 (±0.4)	17.9 (±0.9)	0.7 (±0.1)	0.3 (±0.1)	3.9 (±0)	3.9 (±0.3)

**Table 3:** Contact angle measurements of the Paraloid B-72-treated mockups after aging and after cleaning by NSE-loaded hydrogels (three replicates were involved to get the average values).

Samples	Contact Angles, α (◦)		
	Untreated	With Aged Paraloid B-72	After Cleaning with NSE-Loaded Hydrogels
Marble	81 (±1)	115 (±4)	88 (±3)
Wood	-	108 (±2)	-

## Data Availability

The data presented in this research study are available in the present article and in the related Supplementary Information.

## Supplementary Materials

The following supporting information can be downloaded at: https://www.mdpi.com/article/10.3390/gels12060557/s1, Figure S1: Porosity distribution of S-SB hydrogel; Figure S2: FTIR spectrum of starch biopolymer with hydrogels S-SB and S-Ca.; Figure S3: Raman spectrum of starch biopolymer with hydrogels S-SB and S-Ca.; Figure S4: SEM images of non-lyophilized hydrogel films before (left) and after (right) application processes: (A and B) S-Ca and (C and D) S-SB; Table S1: Moisture properties of newly prepared hydrogels: three replicates were used to calculate the statistical data.; Table S2: Chromatic variations of the Paraloid B-72 treated mock-ups after ageing and after the cleaning with NSE-loaded hydrogels: three replicates were used to calculate the statistical data. Statistical significance for each chromatic coordinate before and after the hydrogel treatment was assessed by paired Student’s *t*-tests; asterisks indicate significant differences (*: *p* < 0.05; **: *p* < 0.01; ***: *p* < 0.001).; Table S3: Contact angle measurements of the Paraloid B-72 treated mock-ups after ageing and after the cleaning with NSE-loaded hydrogels: three replicates were used to calculate the statistical data. For marble substrate, different superscript letters (a, b, c) within the same row indicate statistically significant differences between the experimental conditions (untreated, with aged Paraloid B-72, and after cleaning), assessed by one-way ANOVA followed by Tukey’s post-hoc test (*p* < 0.05).
