# Supplementary material for: Evaluation of Starch-Derived Hydrogel Systems for Artifact-Cleaning Applications
Source: Gels. 2026 Jun 20;12(6):557. doi: 10.3390/gels12060557 (PMC13299439; doi:10.3390/gels12060557)
Supplement: Supplementary file 1 [file gels-12-00557-s001.zip › gels-4342052-supplementary.pdf]

Article

Evaluation of Starch-Derived Hydrogel Systems for Artifact-Cleaning Applications

Nicola Razza [1], Maduka L. Weththimuni [2,*], Matteo Ferretti [2], Alessandro Girella [1,2,3], Barbara Vigani [4], Pietro Galinetto [5] and Maurizio Licchelli [1,2,*]

[1] Research Centre for Cultural Heritage (CISRiC), University of Pavia, via A. Ferrata 3, 27100 Pavia, Italy; nicola.razza01@universitadipavia.it (N.R.); alessandro.girella@unipv.it (A.G.)
[2] Department of Chemistry, University of Pavia, via T. Taramelli 12, 27100 Pavia, Italy; matteo.ferretti02@universitadipavia.it (M.F.)
[3] Centre for Colloid and Surface Science (C.S.G.I.), Pavia Unit, Department of Chemistry, University of Pavia, via T. Taramelli 16, 27100 Pavia, Italy
[4] Department of Drug Sciences, University of Pavia, via T. Taramelli 14, 27100 Pavia, Italy; barbara.vigani@unipv.it (B.V.)
[5] Department of Physics, University of Pavia, via Agostino Bassi, 6, 27100 Pavia, Italy; pietro.galinetto@unipv.it (P.G.)
* Correspondence: madukalankani.weththimuni@unipv.it (M.L.W.); maurizio.licchelli@unipv.it (M.L.)

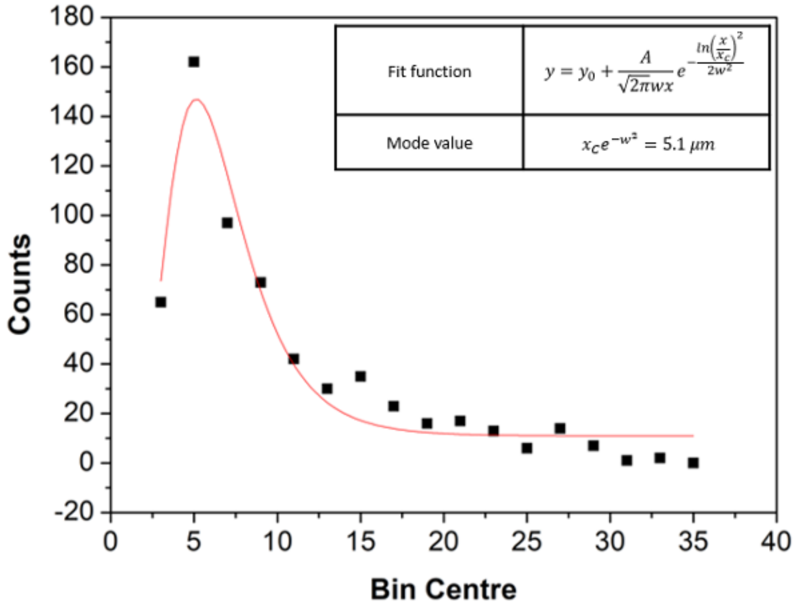

Figure S1. Porosity distribution of S-SB hydrogel.

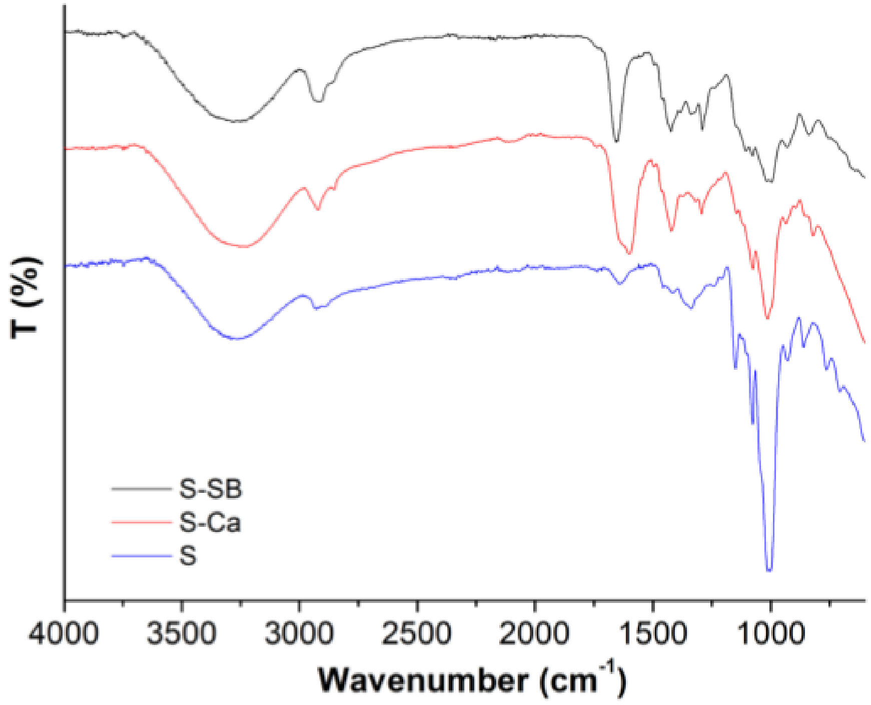

Figure S2. FTIR spectrum of starch biopolymer with hydrogels S-SB and S-Ca.

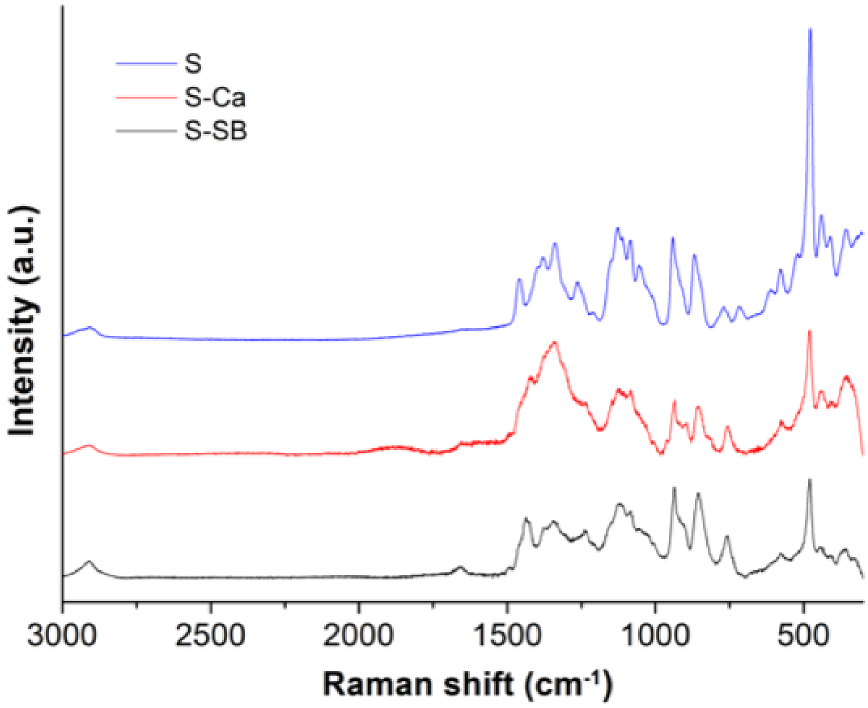

Figure S3. Raman spectrum of starch biopolymer with hydrogels S-SB and S-Ca.

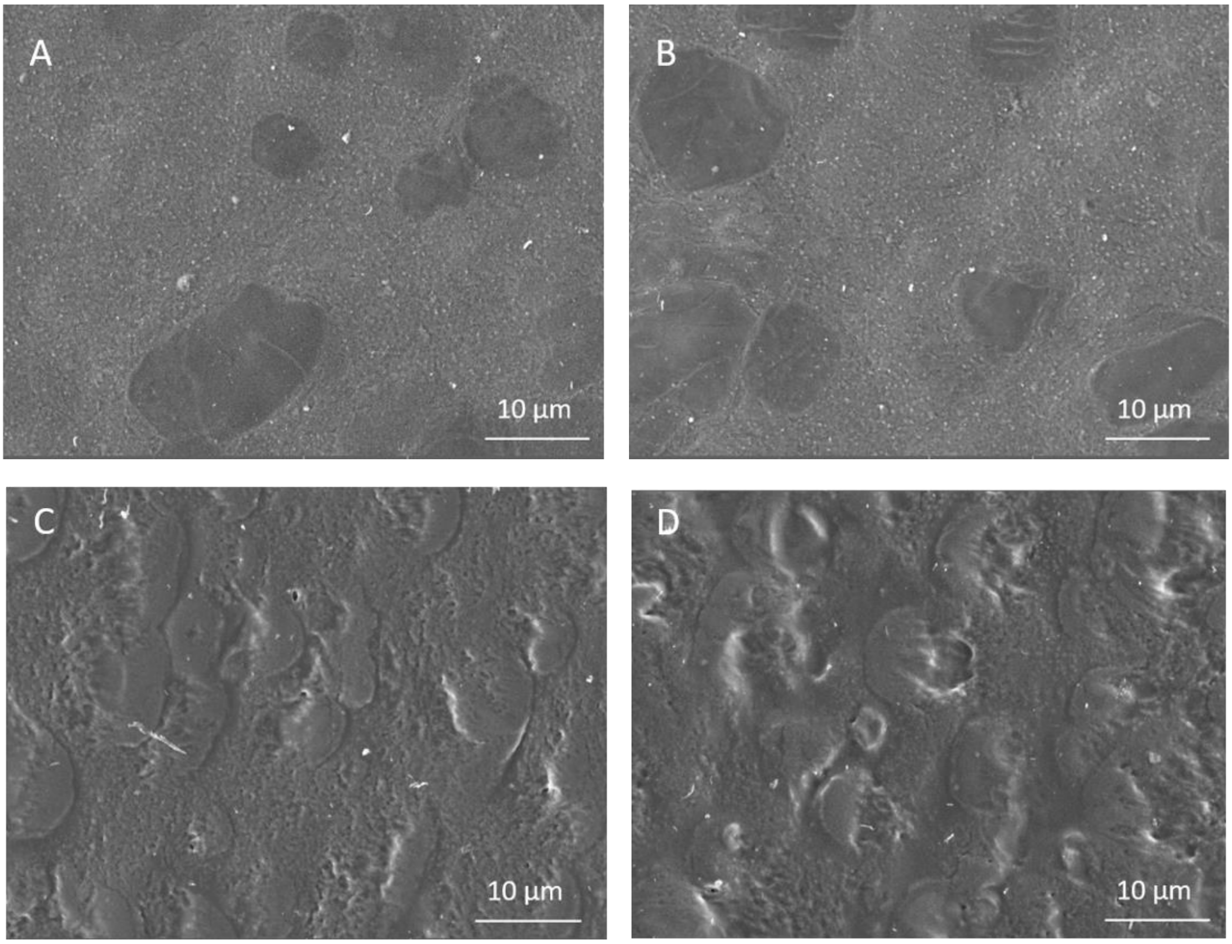

Figure S4. SEM images of non-lyophilized hydrogel films before (left) and after (right) application processes: (A and B) S-Ca and (C and D) S-SB

Table S1. Moisture properties of newly prepared hydrogels: three replicates were used to calculate the statistical data

Gels	Equilibrium water content (EWC, %)	Water releasing capacity (RC, mg/cm^2)
S-Ca	83.3 (±3.1)	11.9 (±2.6)
1	85.6	8.9
2	84.6	12.9
3	79.8	13.8
S-SB	86.3 (±2.8)	16.2 (±0.6)
1	83.1	15.7
2	87.8	16
3	87.9	16.9

Table S2. Chromatic variations of the Paraloid B-72 treated mock-ups after ageing and after the cleaning with NSE-loaded hydrogels: three replicates were used to calculate the statistical data. Statistical significance for each chromatic coordinate before and after the hydrogel treatment was assessed by paired Student's t-tests; asterisks indicate significant differences (*: $p < 0.05$; **: $p < 0.01$; ***: $p < 0.001$).

Samples	With Aged Paraloid B-72				After cleaning with NSE loaded gels			
	ΔL*	Δa*	Δb*	ΔE*	ΔL*	Δa*	Δb*	ΔE*
Marble	-5.3 (±0.2)	-0.1 (±0)	0.5 (±0.2)	5.3 (±0.3)	-1.3 (0.2)***	-0.1 (±0)	0.5 (±0)	1.3 (±0.2)
1	-5.1	-0.1	0.6		-1.1	-0.1	0.5	
2	-5.5	-0.1	0.7		-1.3	-0.1	0.5	
3	-5.3	-0.1	0.3		-1.5	-0.1	0.5	
Wood	-5.7 (±0.3)	0.8 (±0)	5.8 (±0.4)	17.9 (±0.9)	0.7 (±0.1)**	0.3 (±0.1)*	3.9 (±0)*	3.9 (±0.3)
1	-6	0.8	6.2		0.8	0.4	3.9	
2	-5.4	0.8	5.5		0.6	0.2	3.9	
3	-5.7	0.8	5.6		0.7	0.3	3.9	

Table S3. Contact angle measurements of the Paraloid B-72 treated mock-ups after ageing and after the cleaning with NSE-loaded hydrogels: three replicates were used to calculate the statistical data**.** For marble substrate, different superscript letters (a, b, c) within the same row indicate statistically significant differences between the experimental conditions (untreated, with aged Paraloid B-72, and after cleaning), assessed by one-way ANOVA followed by Tukey's post-hoc test ($p < 0.05$).

Samples	Contact angles, α (◦)		
	untreated	With aged Paraloid B-72	After cleaning with NSE loaded gel
**Marble**	81 (±1)^{a}	115 (±4)^{b}	88 (±3)^{c}
1	82	111	85
2	80	115	91
3	81	119	88
**Wood**	-	108 (±2)	-
1	-	110	-
2	-	108	-
3	-	106	-
